# The *PNPLA3* rs738409 G-Allele Associates with Reduced Fasting Serum Triglyceride and Serum Cholesterol in Danes with Impaired Glucose Regulation

**DOI:** 10.1371/journal.pone.0040376

**Published:** 2012-07-05

**Authors:** Nikolaj Thure Krarup, Niels Grarup, Karina Banasik, Martin Friedrichsen, Kristine Færch, Camilla Helene Sandholt, Torben Jørgensen, Pernille Poulsen, Daniel Rinse Witte, Allan Vaag, Thorkild Sørensen, Oluf Pedersen, Torben Hansen

**Affiliations:** 1 The Novo Nordisk Foundation Center for Basic Metabolic Research, Section of Metabolic Genetics, Faculty of Health Sciences, University of Copenhagen, Copenhagen, Denmark; 2 Faculty of Health Sciences, University of Copenhagen, Copenhagen, Denmark; 3 Novo Nordisk A/S, Bagsvaerd, Denmark; 4 Research Centre for Prevention and Health, Glostrup, Denmark; 5 Department of Exercise and Sports Sciences, Copenhagen, Denmark; 6 Steno Diabetes Center, Gentofte, Denmark; 7 Department of Diabetes and Metabolism, Rigshospitalet, Copenhagen, Denmark; 8 Institute of Preventive Medicine, Copenhagen University Hospital, Copenhagen, Denmark; 9 Faculty of Health Sciences, University of Aarhus, Aarhus, Denmark; 10 Faculty of Health Sciences, University of Southern Denmark, Odense, Denmark; Karolinska Insitutet, Sweden

## Abstract

**Background and Aim:**

Non-alcoholic fatty liver disease (NAFLD) is a common condition, associated with hepatic insulin resistance and the metabolic syndrome including hyperglycaemia and dyslipidemia. We aimed at studying the potential impact of the NAFLD-associated *PNPLA3* rs738409 G-allele on NAFLD-related metabolic traits in hyperglycaemic individuals.

**Methods:**

The rs738409 variant was genotyped in the population-based Inter99 cohort examined by an oral glucose-tolerance test, and a combined study-sample consisting of 192 twins (96 twin pairs) and a sub-set of the Inter99 population (*n* = 63) examined by a hyperinsulinemic euglycemic clamp (*n*_total_ = 255). In Inter99, we analyzed associations of rs738409 with components of the WHO-defined metabolic syndrome (*n* = 5,847) and traits related to metabolic disease (*n* = 5,663). In the combined study sample we elucidated whether the rs738409 G-allele altered hepatic or peripheral insulin sensitivity. Study populations were divided into individuals with normal glucose-tolerance (NGT) and with impaired glucose regulation (IGR).

**Results:**

The case-control study showed no associations with components of the metabolic syndrome or the metabolic syndrome. Among 1,357 IGR individuals, the rs738409 G-allele associated with decreased fasting serum triglyceride levels (per allele effect(β) = −9.9% [−14.4%;−4.0% (95% CI)], *p* = 5.1×10^−5^) and fasting total cholesterol (β = −0.2 mmol/l [−0.3;−0.01 mmol/l(95% CI)], *p* = 1.5×10^−4^). Meta-analyses showed no impact on hepatic or peripheral insulin resistance in carriers of the rs738409 G-allele.

**Conclusion:**

Our findings suggest that the G-allele of *PNPLA3* rs738409 associates with reduced fasting levels of cholesterol and triglyceride in individuals with IGR.

## Introduction

Non-alcoholic fatty liver disease (NAFLD) is defined as the deposition of fat in hepatocytes exceeding 5–10% of the liver-weight, not caused by excessive alcohol consumption [1]. The disease is becoming increasingly prevalent and is estimated to involve 30% of the general population in the U.S.A. [2]. NAFLD is often present in obese individuals and liver fat content is linearly correlated to components of the metabolic syndrome, e.g. increased fasting plasma glucose- and fasting serum triglyceride-levels and increased waist circumference [3], [4]. Furthermore, accumulation of fat in the liver is associated with hepatic insulin resistance [5], [6]. Hepatic content of triacylglycerols (TAG) is derived from uptake of circulating albumin-bound fatty acids, very-low density lipoprotein (VLDL) and chylomicron remnants and increased circulating levels of glucose can act as substrate for *de novo* lipogenesis and may inhibit fatty acid oxidation [7]–[9]. Regulation of hepatic TAG synthesis and degradation is influenced by insulin levels and by circulating glucose levels via the liver X-receptor (LXR) which regulates carbohydrate response element binding protein (ChREBP), the sterol response element binding protein-1c (SREBP-1c) and downstream enzymes involved in fatty acid and TAG synthesis [10].

Recent investigations have examined whether common genetic variants associate with NAFLD [11]–[13]. These studies identified the G-allele of rs738409 in *PNPLA3*, changing the amino-acid isoleucine to methionine at location 148 (I148M) to associate with NAFLD on a genome-wide significant level (*p*-value <5×10^−8^). Interestingly, although association with hepatic lipid accumulation was validated, the variant did not affect VLDL-, LDL-, HDL-, total-cholesterol levels, insulin resistance or circulating glucose levels [8], [14]–[18].

We aimed at examining the effect of the rs738409 on traits related to the metabolic syndrome in individuals in the population based Inter99 study. Moreover, as lipogenesis is influenced by availability of glucose [7]–[9] and *PNPLA3* expression is influenced by glucose-levels [18] we aimed to examine whether the effect of *PNPLA3* rs738409 on metabolic traits is influenced by hyperglycaemia.

## Methods

### Ethnicity and ethical statement

All participants were Danes by self-report and written informed consent was obtained from all individuals before participation. The studies were approved by the regional Ethical Committee of Copenhagen and were conducted in accordance with the principles of the Helsinki Declaration.

### Study populations

A case-control study of the metabolic syndrome defined according to the 1999 WHO criteria [19] was performed in the Inter99 population (*n* = 5,847) where genotype was available. The Inter99 population is a randomised multi-factorial lifestyle intervention study for prevention of ischemic heart disease (ClinicalTrials.gov ID-no: NCT00289237). Control individuals (*n* = 1,691) were defined as not having any of the components comprised in the WHO-defined criteria of the metabolic syndrome. Case-individuals with a HOMA-IR value in the highest quartile of the population distribution were defined as having insulin resistance (*n* = 1,497). Cases with dyslipidemia (*n* = 1,526) were defined as having serum TAG >1.7 mM and/or HDL-cholesterol <0.9 mM for men and <1 mM for women or if they were treated with lipid-lowering agents. Cases with hypertension (*n* = 2,383) were defined as having a systolic blood pressure above 140 mmHg and/or diastolic blood pressure above 90 mmHg or if they were treated with antihypertensive medication. Obese cases (*n* = 2,637) were defined either by a BMI above 30 kg/m^2^ or a waist-to-hip ratio above 0.9 for men and above 0.85 for women. Cases with albuminuria (*n* = 170) were defined as having an albumin/creatinine ratio above 30 mg/g. Individuals can belong to more than one group.

Analysis of quantitative metabolic traits related to NAFLD was also performed in the population-based Inter99 cohort of individuals of Danish nationality (*n* = 5,663). The cohort was stratified in individuals with normal glucose-tolerance (NGT, *n* = 4,306) and individuals with impaired glucose regulation (IGR, *n* = 1,357) according to WHO-criteria [19]. Individuals with known type 2 diabetes (T2D) receiving oral antidiabetic or insulin treatment (*n* = 118) and individuals receiving lipid-lowering treatment (*n* = 68) were excluded from the analyses of quantitative metabolic traits. Individuals with IGR included individuals with 1) IFG (*n* = 459), 2) IGT (*n* = 644) and 3) newly-diagnosed untreated T2D (*n* = 254). All participants were examined by a standardised questionnaire and interview, physical examination and blood sampling before and during a standardized 75g OGTT.

*In vivo* hepatic and peripheral insulin sensitivity was measured by euglycemic hyperinsulinemic clamps using tritiated glucose in two separate study populations: 1) 98 young (22–31 years) and elderly (57–66 years) monozygotic and same-sex dizygotic twin pairs (YOND), including 149 twins with NGT, 23 twins with IFG, 21 with IGT and 3 with previously undiagnosed diabetes [20]–[22] and 2) a sub-set of the Inter99 population (*n* = 63) including 18 participants with NGT, 17 with isolated IFG and 28 with isolated IGT [23]. Meta-analyses included a study sample of 183 NGT and 68 IGR individuals in total.

### Biochemical and anthropometric measures

In the Inter99 study population, height and body weight were measured in light indoor clothing without shoes. BMI was calculated as weight (kg)/(height [m])^2^. All blood samples were obtained after a 12 hour overnight fast and Inter99 participants underwent an OGTT. Insulin sensitivity was estimated by the homeostasis model assessment of insulin resistance (HOMA-IR; (fasting plasma glucose [mmol/l] ×fasting serum insulin [pmol/l])/22.5) [24]. Levels of LDL cholesterol were calculated as described earlier [25].

The clinical examinations of the YOND cohort (n = 192) and the Inter99 subset (n = 63) have previously been described in detail [20], [21], [23]. Individuals without genotype information were excluded (n = 4). In brief, peripheral insulin sensitivity was in both populations examined by a 2 hour euglycemic-hyperinsulinemic clamp (40 mU m^−2^ min^−1^). A primed constant continuous infusion of [3^−3^H]-tritiated glucose (bolus 22 μCi, 0.22 μCi min^−1^) was initiated at 0 min and continued throughout the clinical investigation (basal period [120 min] and clamp period [120 min]). Steady-state was defined as the last 30 min of the basal and insulin-stimulated periods [22]. Peripheral insulin sensitivity was calculated as the rate of glucose disappearance during insulin stimulation (Rd clamp) using the non-steady-state equation [21]. Hepatic insulin resistance was estimated as basal endogenous glucose production multiplied by fasting serum insulin concentration [26].

### Genotyping

For all the included study samples, genotyping of the *PNPLA3* rs738409 was performed using KASPar SNP Genotyping (KBioscience, Hoddesdon, UK). Genotyping success rate was 96.7% and the error rate was 0.92% as estimated from 1,187 duplicate samples. Genotype distribution obeyed Hardy-Weinberg equilibrium in the analysed study samples (*p*>0.05).

### Statistical analysis

In the case-control study of the metabolic syndrome and related traits, we used logistic regression to examine differences in genotypes assuming an additive model adjusting for age and gender. The analysis consisted of pair-wise analysis of genotype frequencies between metabolic syndrome related traits and control individuals (with none of the characteristics of metabolic derangement). We assumed an additive genetic model, based on earlier findings of the effect of the variant. In the Inter99 study population, a general linear model was used to test anthropometric, OGTT-derived and biochemical traits. All analyses were adjusted for sex, age and BMI. Serum TAG levels, HOMA-IR and estimates of hepatic and peripheral insulin sensitivity were logarithmically transformed before analysis. Interaction analyses between genotype and glucose tolerance status (divided into two groups; NGT and IGR), obesity status (BMI below 25, BMI from 25 to 30 and BMI over 30) and OGTT-derived glucose levels (quantitative outcomes) on fasting TAG and total cholesterol levels were performed using linear regression with a multiplicative interaction term. Statistical analyses were performed using RGui, version 2.7.2 (available at http://www.r-project.org).

In the YOND study sample and the Inter99 subset, statistical tests were performed in SAS (version 9.1, SAS Institute, Cary, NC) also using the linear regression model adjusting for age, sex, and BMI. All response variables were log-transformed before analysis. Effect sizes of log-transformed variables (with β >0.05) were calculated as 100×e^β^-1. Fixed-effect meta-analyses were performed to increase power to detect an association with estimates of hepatic (Hepatic IR basal) and peripheral insulin resistance (Rd clamp). We used effect size estimates and standard errors derived from analyses of the mentioned traits where the effect of the genetic variant was estimated as the effect per allele. Weight of studies was estimated using inverse variance assuming fixed effects. Heterogeneity was measured by Q-statistics. Multiple regression analyses using the *proc mixed* procedure in SAS allowed for adjustment of twin pair and zygosity status and other contributing variables [27].

A *p*-value below 0.05 was considered significant. No correction for multiple testing (Bonferroni) was performed. Power analysis was based on 1000 simulations. We used empirical variance of the observed traits in the Inter99 cohort to simulate phenotypes from a normal distribution. Statistical power analysis showed approximately 50% statistical power to detect an association with measures of insulin resistance in the small combined study-sample (n_total_ = 251) if the true allelic effect size is 10% on the level of a given trait. Conversely, power estimates on the quantitative trait analysis in Inter99 revealed more robust statistical power (>80%) to detect association assuming a minor allele frequency of 22% and an allele-dependent effect size of 8% on the level of a given trait.

## Results

### Case- control study of the metabolic syndrome

A case-control study showed no associations with components of the metabolic syndrome or the metabolic syndrome (Table 1).

**Table 1:** Genotype and allele frequency of rs738409 among individuals with traits related to the metabolic syndrome in Inter99.

	Control individuals	Impaired glucose regulation	Insulin resistance	Hypertension	Dyslipidemia	Indices of obesity	Micro-albuminuria	One or more components of the metabolic syndrome	The metabolic syndrome
N Men/Women	1,691 (493/1198)	1,462 (877/585)	1,497 (830/667)	2,383 (1413/970)	1,526 (983/543)	2,637 (1792/845)	170 (76/94)	4,085 (2380/1705)	1,306 (882/424)
CC (%)	1010 (59.7)	911 (62.3)	882 (58.9)	1440 (60.4)	931 (61)	1597 (60.6)	102 (60)	2458 (60.2)	800 (61.3)
CG (%)	593 (35.1)	480 (32.8)	527 (35.2)	812 (34.1)	517 (33.9)	912 (34.6)	58 (34.1)	1407 (34.4)	440 (33.7)
GG (%)	88 (5.2)	71 (4.9)	88 (5.9)	131 (5.5)	78 (5.1)	128 (4.9)	10 (5.9)	220 (5.4)	66 (5.1)
G-allele frequency [%(95% CI)]	22.7 (21.3–24.2)	21.3 (19.8–22.8)	23.5 (22–25)	22.5 (21.4–23.7)	22.1 (20.6–23.6)	22.1 (21–23.3)	22.9 (18.6–27.8)	22.6 (21.7–23.5)	21.9 (20.3–23.5)
Allele frequency model OR (95% CI)		0.92 (0.81–1.04)	1.04 (0.93–1.17)	0.99 (0.89–1.1)	0.96 (0.85–1.08)	0.97 (0.87–1.07)	1.01 (0.77–1.33)	0.99 (0.9–1.09)	0.95 (0.84–1.08)
p_freq_		0.17	0.49	0.83	0.51	0.53	0.95	0.88	0.45
Additive model OR (95% CI)		0.87 (0.74–1.02)	1.07 (0.91–1.26)	0.97 (0.85–1.11)	0.99 (0.85-1-16)	0.96 (0.82–1.12)	0.98 (0.72–1.33)	1.02 (0.91–1.14)	0.88 (0.77–1.09)
p_add_		0.09	0.39	0.7	0.94	0.58	0.89	0.74	0.25

The table presents a case-control study of the association of the rs738409 with the metabolic syndrome. It includes 5,847 individuals from the Inter99 cohort. Individuals may have more than one trait. The metabolic syndrome was defined according to the 1999 WHO criteria. Control individuals (*n* = 1,691) were defined as not having any of the components comprised in the WHO-defined criteria of the metabolic syndrome. Case-individuals with IGR were defined as mentioned in the main text. Individuals with a HOMA-IR value in the highest quartile of the population distribution were defined as having insulin resistance (*n* = 1,497). Cases with dyslipidemia (*n* = 1,526) were defined as having serum TAG >1.7 mM and/or HDL-cholesterol <0.9 mM for men and <1 mM for women or treated with lipid-lowering agents. Cases with hypertension (*n* = 2,383) were defined as having a systolic blood pressure above 140 mmHg and/or diastolic blood pressure above 90 mmHg or treated with antihypertensive medication. Obese cases (*n* = 2,637) were defined either by a BMI above 30 kg/m^2^ or a waist-to-hip ratio above 0.9 for men and above 0.85 for women. Cases with albuminuria (*n* = 170) were defined as having an albumin/creatinine ratio above 30 mg/g. Values are means ± SD. Traits were defined as according to the 1999 WHO-defined metabolic syndrome components. Individuals may have more than one trait. P-values were adjusted for age, sex and BMI.

### Quantitative trait analysis of traits related to metabolic disease

Among 4,306 individuals with NGT, the rs738409 G-allele was associated with fasting plasma glucose levels (β = −0.4% [−0.7%; −0.01% (95% CI)], *p* = 0.04). Among 1,357 individuals with IGR, the minor, methionine-coding G-allele was significantly associated with decreased fasting levels of both serum TAG (per allele effect (β) [95% CI]  = −9.2% [−14.4%;−4.0%], *p* = 5.1×10^−5^) and total serum cholesterol (β = −0.2 mmol/l [−0.3;−0.1], *p* = 1.5×10^−4^) (Table 2). In an interaction analysis between genotype and glucose-tolerance status on the two associating lipid traits, we found statistically significant interactions between the rs738409 G-allele and glucose tolerance status on TAG and cholesterol (*p* = 2×10^−4^ and *p* = 8×10^−5^, respectively Table 2). In individuals with IGR, no other associations were observed between the rs738409 genotype and metabolic disease-associated traits.

**Table 2:** Quantitative traits among individuals from the Inter99 cohort according to *PNPLA3* rs738409 genotype.

	CC		CG			GG		Effect (95%CI)		p*_add_*	p*_interaction_*		
**Normal glucose tolerance (** ***n*** ** = 4,306)**													
*N* (men/women)		2559 (1161/1398)	1512 (709/803)			235 (115/120)							
Age (years)		45±8	45±8			45±8							
Fasting plasma glucose (mmol/l)		5.3±0.4	5.3±0.4			5.3±0.4		−0.004 (−0.007–−0.0001)		0.04			
2-hour plasma glucose (mmol/l)		5.5±1.1	5.5±1.1			5.4±1.2		−0.04% (−1.5%–0.6%)		0.4			
Fasting serum insulin (pmol/l)		37±23	37±23			37±22		−0.05 (−0.02–0.03)		0.7			
BMI (kg/m^2^)		25.5±4.1	25.5±4.1			24.9±3.6		−0.2 (−0.4–−0.003)		0.05			
Waist (cm)		84±12	84±12			83±11		−0.6 (−0.3–0.2)		0.7			
HOMA-IR (mmol×pmol/l)		8.9±5.7	8.9±5.6			8.8±5.4		0.002 (−0.02–0.03)		0.9			
Total serum cholesterol (mmol/l)		5.4±1	5.4±1			5.4±1.1		0.0002 (−0.05–0.05)		1			
HDL (mmol/l)		1.5±0.4	1.4±0.4			1.5±0.4		−0.01 (−0.03–0.007)		0.2			
LDL (mmol/l)		3.4±1	3.5±0.9			3.5±1		0.04 (−0.02–0.1)		0.2			
VLDL (mmol/l)		0.5±0.3	0.6±0.3			0.5±0.3		−0.008 (−0.03–0.01)		0.4			
Serum triglycerides (mmol/l)		1.2±1	1.2±0.8			1.1±0.6		−1.4% (−3.6%–0.009%)		0.2			
**Impaired glucose regulation (** ***n*** ** = 1,357)**													
*N *(men/women)		859 (514/345)		437 (263/174)	61 (40/21)								
Age (years)		49±7		49±8	49±7								
Fasting plasma glucose (mmol/l)		6.3±1.3		6.2±1	6.3±1.4		−0.3% (−1.6%–1%)		0.7				
2-hour plasma glucose (mmol/l)		8.4±2.8		8.5±2.9	8.4±3.4		0.1% (−2.8%–3%)		0.9				
Fasting serum insulin (pmol/l)		55±35		56±36	64±35		3% (−1.6%–7.8%)		0.2				
BMI (kg/m^2^)		28.2±4.9		28.3±5.2	30.1±7		0.43 (−0.05–0.9)		0.08				
Waist (cm)		93±13		93±14	97±16		−0.14 (−0.65–0.37)		0.6			0.7	
HOMA-IR (mmol×pmol/l)		15.5±11.2		16±11.8	18.1±10.5		0.03 (−0.02–0.08)		0.3			0.3	
Total serumcholesterol (mmol/l)		5.9±1.1		5.7±1.1	5.6±1.1		−0.2 (−0.3–−0.1)		1.5×10−4			8×10−5	
HDL (mmol/l)		1.4±0.4		1.4±0.4	1.3±0.4		0.005 (−0.03–0.04)		0.8			0.4	
LDL (mmol/l)		3.8±1		3.6±1.1	3.7±0.9		−0.13 (−0.27–−0.01)		0.08			0.02	
VLDL (mmol/l)		0.8±0.4		0.7±0.4	0.8±0.4		−0.04 (−0.09–0.01)		0.16			0.2	
Serum triglycerides (mmol/l)		1.9±1.6		1.6±1.1	1.5±0.8		−9.9%(−14.4%–−4.0%)		5.1×10−5			2×10−4	

Numbers are mean ± SD. Analyses were made using an additive model. P-values were adjusted for age, sex and BMI. Individuals from the Inter99 receiving oral antidiabetic or lipid-lowering treatment (*n* = 186) were excluded. Effects are changes in units unless calculated on log-transformed traits (change in percent).

Interaction of genotype with quantitative glucose traits and BMI was subsequently analyzed (Table S1). Interaction was observed between genotype and 2-hour OGTT plasma glucose values for fasting serum TAG levels (*p* = 0.009, Table S1). No interaction with BMI was observed.

### Meta-analysis of hepatic and peripheral insulin resistance

To investigate whether the NAFLD risk rs738409 G-allele was associated with altered insulin resistance in either the liver or peripheral tissues, we performed a meta-analysis based on estimates of hepatic and peripheral insulin resistance obtained from a euglycaemic hyperinsulinaemic clamp examination in NGT (*n* = 183) and IGR (*n* = 68) individuals (Supplemental Figure S1). For NGT individuals, a nominal increase in peripheral insulin sensitivity in rs738409 G-allele carriers measured by insulin-stimulated glucose-disposal (β = 9.6% [0.5%;18.8%], *p* = 0.04) was observed. No association between the rs738409 genotype and peripheral insulin sensitivity was found in IGR individuals. Furthermore, hepatic insulin resistance was not associated with the rs738409 genotype in either NGT or IGR individuals.

## Discussion

This study reports an association of the *PNPLA3* rs738409 NAFLD risk G-allele with decreased fasting levels of serum TAG and serum cholesterol in individuals with IGR but not among individuals with NGT. Analysis of interaction between genotype and glucose tolerance status or OGTT derived 2 hour glucose levels revealed significant interactions. The rs738409 G-allele conferred reduced serum TAG and total cholesterol levels among individuals with elevated glucose levels. In a population-based sample of 5,847 Danes we found no association with the metabolic syndrome. In NGT individuals, we observed a nominal significant association with decreased fasting plasma glucose levels. Furthermore, the rs738409 G-allele nominally associated with increased peripheral insulin sensitivity, estimated by a euglycemic hyperinsulinemic clamp. Our findings indicate that the effect of the rs738409 G-allele on fasting lipid levels is unmasked in a state of hyperglycaemia.

Changes in fasting circulating levels of TAG are believed to be conferred endogenously by changes in levels of circulating VLDL, stemming from hepatic secretion. Interestingly, recent findings of an association of *GCKR* variation with increased VLDL particle concentrations [28], [29] suggests that enhanced glycolytic flux leads to increased levels of *de novo* TAG and cholesterol synthesis.

Functional studies of *PNPLA3* I148M-substituted cells reveal a plausible mechanistic explanation for the decreased lipid levels observed in IGR G-allele carriers of rs738409. *PNPLA3* encodes adiponutrin, a member of the calcium-independent phospholipase A2 family, having triacylglycerol hydrolase activity and possibly acylglycerol transacylase activity [6]. The enzyme is regulated by insulin levels and in animal models the mRNA levels of *PNPLA3* are low in the fasting state but rise significantly during carbohydrate feeding [18]. Furthermore, a study of *in vitro* assays of wild-type *PNPLA3* and I148M-substituted *PNPLA3* revealed a marked reduction in hydrolysis of intracellular stores of TAGs in hepatocytes [30]. We suggest that IGR rs738409 G-allele carriers have an increased glycolytic flux and generation of precursors for synthesis of lipid molecules, e.g. TAGs and cholesterol. Individuals with decreased hepatic lipolysis conferred by the I148M variant have a decreased hepatic release of TAG and cholesterol to the circulation and they may have a relatively benign form of elevated intrahepatocellular concentration of TAG, which does not associate with the metabolic syndrome.

Association of the rs738409 G-allele with decreased fasting levels of TAG has only been observed in smaller study populations (*n* = 144) of individuals selected for NAFLD and steatohepatitis of Japanese, Indian, Malayan and Chinese ancestry [31], [32] as well as in 516 American non-Hispanic individuals [33]. All individuals had severe NAFLD. Subsequent analyses in larger cohorts (n = 19,840) who were not ascertained for fatty liver disease (and were not stratified on the degree of glucose intolerance) did not reveal association with lipid levels [33]. Another study has shown an interaction between the rs738409 G-allele and a reduction in apoB-containing lipoproteins in 23,274 individuals from eight independent West-Eurasian study populations and one population from Utah, U.S.A [34].

In NGT individuals, careful interpretation must be taken in relation to the nominal association with decreased levels of fasting plasma glucose for the risk G-allele. The nominal significant difference between genotype groups for fasting plasma glucose in NGT individuals is only significant when adjusting for BMI, and likely a chance finding.

In smaller study samples, the rs738409 G-allele has been associated with decreased insulin resistance measured by HOMA-IR [14], [17], [35]. In a meta-analysis of relatively small but carefully phenotyped study samples, we find increased insulin-stimulated glucose disposal in NGT individuals as estimated by the hyperinsulinaemic euglycaemic clamp. However, careful appraisal of this finding must be adopted as the association is not corrected for multiple testing.

### Conclusion

The present study points to an association of the common rs738409 minor G-allele of *PNPLA3* with decreased levels of fasting serum TAG and total serum cholesterol in individuals with impaired glucose tolerance. Future studies should stratify study participants according to glucose-tolerance status (i.e. normoglycaemia and hyperglycaemia) or include information on 2-hour OGTT plasma glucose levels to assess the effect of the *PNPLA3* rs738409 G-allele on lipid levels.

## Supporting Information

Figure S1 **Meta-analyses of insulin resistance measures.** Meta-analyses of 251 individuals of YOND (*n* = 188) and Inter99 sub-set (*n* = 63) stratified into individuals with normal glucose-tolerance (NGT, *n* = 165 in YOND; *n* = 18 in I99 sub-set) or impaired glucose regulation (IGR, *n* = 23 in YOND; *n* = 45 in I99 sub-set). Effect sizes for the G-allele are in percentages and standard errors were obtained from analyses done separately in the study samples. The values were combined using the inverse variance method. Black squares are effects in single studies according to weight in the meta-analysis. Black diamonds are the combined change in either hepatic insulin resistance (Basal Hepatic IR) or peripheral insulin resistance (Rd clamp). **A** shows hepatic insulin resistance(IR) in NGT individuals (Combined effect size [95% CI]  = −13.3% [−28.6 to 2.1%], *p* = 0.09), **B** shows peripheral insulin resistance in NGT individuals (Combined effect size [95% CI]  = 9.7% [0.05% to 18.8%], *p* = 0.04), **C** shows hepatic insulin resistance in IGR individuals (Combined effect size [95% CI]  = 3.3% [−12.5% to 19.1%], *p* = 0.7), **C** shows peripheral insulin resistance in IGR individuals (Combined effect size [95% CI]  = 1.0% [−7% to 9%], *p* = 0.8).
(DOCX)

Table S1 **Interaction analyses of rs738409 genotype and interaction variables.** The table shows *p*-values and effect estimates for interaction of glucose-tolerance, glucose-levels or BMI with genotype on triglyceride and total cholesterol levels. Effect estimates are percentage change in levels of fasting serum triglyceride or changes in millimoles per liter for total cholesterol levels. The strongest interaction is seen between glucose tolerance and genotype on both lipid traits. Interaction is also seen between genotype and levels of 2-hour glucose after an OGTT. All *p*-values are adjusted for age and gender. Glucose-related *p*-values are additionally adjusted for BMI, and BMI was adjusted for glucose-tolerance. The BMI variable is categorized into lean (BMI<25), overweight (BMI = 25–30) and obese individuals (BMI>30).
(DOCX)
