# Correction: The *PNPLA3* rs738409 G-Allele Associates with Reduced Fasting Serum Triglyceride and Serum Cholesterol in Danes with Impaired Glucose Regulation

**DOI:** 10.1371/annotation/a8523212-5e34-4475-93d3-9e90021ebb03

**Published:** 2012-09-19

**Authors:** Nikolaj Thure Krarup, Niels Grarup, Karina Banasik, Martin Friedrichsen, Kristine Færch, Camilla Helene Sandholt, Torben Jørgensen, Pernille Poulsen, Daniel Rinse Witte, Allan Vaag, Thorkild I.A. Sørensen, Oluf Pedersen, Torben Hansen

The eleventh author's name was spelled incorrectly. The correct name is: Thorkild I.A. Sørensen.

