# Supplementary material for: The PNPLA3 rs738409 G-Allele Associates with Reduced Fasting Serum Triglyceride and Serum Cholesterol in Danes with Impaired Glucose Regulation
Source: PLoS One. 2012 Jul 5;7(7):e40376. doi: 10.1371/journal.pone.0040376 (PMC3390392; doi:10.1371/journal.pone.0040376)
Supplement: Figure S1 — Meta-analyses of insulin resistance measures. Meta-analyses of 251 individuals of YOND (n = 188) and Inter99 sub-set (n = 63) stratified into individuals with normal glucose-tolerance (NGT, n = 165 in YOND; n = 18 in I99 sub-set) or impaired glucose regulation (IGR, n = 23 in YOND; n = 45 in I99 sub-set). Effect sizes for the G-allele are in percentages and standard errors were obtained from analyses done separately in the study samples. The values were combined using the inverse variance method. Black squares are effects in single studies according to weight in the meta-analysis. Black diamonds are the combined change in either hepatic insulin resistance (Basal Hepatic IR) or peripheral insulin resistance (Rd clamp). A shows hepatic insulin resistance(IR) in NGT individuals (Combined effect size [95% CI] = −13.3% [−28.6 to 2.1%], p = 0.09), B shows peripheral insulin resistance in NGT individuals (Combined effect size [95% CI] = 9.7% [0.05% to 18.8%], p = 0.04), C shows hepatic insulin resistance in IGR individuals (Combined effect size [95% CI] = 3.3% [−12.5% to 19.1%], p = 0.7), C shows peripheral insulin resistance in IGR individuals (Combined effect size [95% CI] = 1.0% [−7% to 9%], p = 0.8). (DOCX) [file pone.0040376.s001.docx]

# Figure S1 Meta-analyses of insulin resistance measures
