# Supplementary material for: The PNPLA3 rs738409 G-Allele Associates with Reduced Fasting Serum Triglyceride and Serum Cholesterol in Danes with Impaired Glucose Regulation
Source: PLoS One. 2012 Jul 5;7(7):e40376. doi: 10.1371/journal.pone.0040376 (PMC3390392; doi:10.1371/journal.pone.0040376)
Supplement: Table S1 — Interaction analyses of rs738409 genotype and interaction variables. The table shows p-values and effect estimates for interaction of glucose-tolerance, glucose-levels or BMI with genotype on triglyceride and total cholesterol levels. Effect estimates are percentage change in levels of fasting serum triglyceride or changes in millimoles per liter for total cholesterol levels. The strongest interaction is seen between glucose tolerance and genotype on both lipid traits. Interaction is also seen between genotype and levels of 2-hour glucose after an OGTT. All p-values are adjusted for age and gender. Glucose-related p-values are additionally adjusted for BMI, and BMI was adjusted for glucose-tolerance. The BMI variable is categorized into lean (BMI<25), overweight (BMI = 25–30) and obese individuals (BMI>30). (DOCX) [file pone.0040376.s002.docx]

| Table S1 Interaction analyses of rs738409 genotype and interaction variables | | | | |
| --- | --- | --- | --- | --- |
| **Interaction variable** | Fasting serum triglyceride | | Fasting total cholesterol | |
|  | β (SEM) | *p*_interaction_ | β (SEM) | *p*_interaction_ |
| NGT vs. IGR | - 0.2 (0.07) | 0.0002 | - 0.21 (0.05) | 8x10^-5^ |
| Fasting plasma glucose | - 4.4x10^-3^ (1.7x10^-2^) | 1 | - 0.03 (0.02) | 0.2 |
| 2 hour plasma glucose | - 0.03 (0.009) | 0.009 | - 0.02 (0.01) | 0.1 |
| BMI | - 0.03 (0.04) | 0.6 | - 0.04 (0.03) | 0.2 |
